# The Relationship between Protein Intake and Source on Factors Associated with Glycemic Control in Individuals with Prediabetes and Type 2 Diabetes

**DOI:** 10.3390/nu12072031

**Published:** 2020-07-08

**Authors:** Neda S. Akhavan, Shirin Pourafshar, Sarah A. Johnson, Elizabeth M. Foley, Kelli S. George, Joseph Munoz, Shalom Siebert, Elizabeth A. Clark, Raedeh Basiri, Robert C. Hickner, Negin Navaei, Cathy W. Levenson, Lynn B. Panton, Bruce P. Daggy, Bahram H. Arjmandi

**Affiliations:** 1 Department of Nutrition, Food and Exercise Sciences, Florida State University, Tallahassee, FL 32304, USA; nsa08@my.fsu.edu (N.S.A.); ef15c@my.fsu.edu (E.M.F.); jm12t@my.fsu.edu (J.M.); ss17u@my.fsu.edu (S.S.); eac15h@my.fsu.edu (E.A.C.); rb14e@my.fsu.edu (R.B.); rhickner@fsu.edu (R.C.H.); lpanton@fsu.edu (L.B.P.); bruce.daggy@gmail.com (B.P.D.); 2 Center for Advancing Exercise and Nutrition Research on Aging, Florida State University, Tallahassee, FL 32304, USA; sp8ds@hscmail.mcc.virginia.edu (S.P.); kelli.george@mail.wvu.edu (K.S.G.); negin.navaei@life.edu (N.N.); 3 Department of Medicine, Division of Nephrology, University of Virginia, Charlottesville, VA 22903, USA; 4 Department of Food Science and Human Nutrition, Colorado State University, Fort Collins, CO 80526, USA; sarah.johnson@colostate.edu; 5 Division of Animal and Nutritional Sciences, West Virginia University, Morgantown, WV 26506, USA; 6 Department of Nutrition, Life University, Marietta, GA 30060, USA; 7 Department of Biomedical Sciences and Program in Neuroscience, Florida State University, Tallahassee, FL 32304, USA; cathy.levenson@med.fsu.edu; 8 Institute for Successful Longevity, Florida State University, Tallahassee, FL 32304, USA

**Keywords:** metabolic syndrome, protein source, cardiovascular risk factors, RDA, diabetes

## Abstract

Type 2 diabetes (T2D) is a major contributor to morbidity and mortality largely due to increased cardiovascular disease risk. This study examined the relationships among protein consumption and sources on glycemic control and cardiovascular health in individuals with prediabetes and T2D. Sixty-two overweight or obese participants with prediabetes or T2D, aged 45–75 years were stratified into the following three groups based on protein intake: <0.8 g (gram)/kg (kilogram) body weight (bw), ≥0.8 but <1.0 g/kg bw, and ≥1.0 g/kg bw as below, meeting, and above the recommended levels of protein intake, respectively. Body mass, body mass index (BMI), hip circumference (HC), waist circumference (WC), lean mass, and fat mass (FM) were significantly higher in participants who consumed below the recommended level of protein intake as compared with other groups. Higher animal protein intake was associated with greater insulin secretion and lower triglycerides (TG). Total, low-density, and high-density cholesterol were significantly higher in participants who met the recommended protein intake as compared with the other groups. These data suggest that high protein consumption is associated with lower BMI, HC, WC, and FM, and can improve insulin resistance without affecting lipid profiles in this population. Furthermore, higher intake of animal protein can improve β-cell function and lower plasma TG.

## 1. Introduction

The incidence of type 2 diabetes (T2D) continues to increase in the United States (USA), with approximately 30.3 million Americans (9.4% of the population) having diabetes and 84 million having prediabetes. Treatment of T2D and associated complications creates a large economic burden; $327 billion were spent, in 2017, for medical costs and lost work and productivity due to T2D [1]. The majority of individuals with T2D and prediabetes are not aware of having this condition until symptoms develop [2]. It is estimated that 70% of those with prediabetes will progress to T2D in their life [3,4]. T2D is a metabolic disease characterized by the body’s resistance to insulin action after a meal, causing hyperglycemia [5]. There are many risk factors contributing to the development of prediabetes and T2D including genetic, metabolic, and environmental factors. Information gained by examination of modifiable risk factors such as weight gain, physical inactivity, or diet can be used to prevent the progression or development of prediabetes and T2D [6]. This is especially critical for prediabetic populations as the implementation of lifestyle interventions can prevent the disease from progressing and can prevent the need for pharmaceutical intervention.

Cardiovascular disease (CVD) is the leading cause of morbidity and mortality for people with diabetes in the USA [7]. Both CVD and T2D have common modifiable risk factors such as obesity, hypertension, and dyslipidemia, and many individuals with T2D have had these risk factors for many years prior to the development of insulin resistance [7,8,9,10,11,12]. The Centers for Disease Control and Prevention has estimated that over two-thirds of adults in the USA are overweight or obese [13]. Increases in adipose tissue have been shown to contribute to dysfunctional processes in the body including inflammation/inflammatory responses, blood pressure, and macronutrient metabolism [9]. Additionally, the localization of adipose tissue has been shown to be an important risk factor for the development of T2D, for example, in a meta-analysis by Kodama et al. [8], waist circumference (WC) had a stronger correlation with the development of diabetes as compared with body mass index (BMI). In addition to obesity and central adiposity, high blood pressure is another common modifiable risk factor observed in this population, in which over 60% of individuals with T2D had hypertension [10]. Prevention and treatment of this condition are critical to prevent the development of macrovascular (heart attack and stroke) and microvascular (nephropathy, retinopathy, and neuropathy) complications [14]. The development of macrovascular complications in individuals with T2D can be due to disturbances in lipid metabolism which can occur years prior to the development of T2D, due to insulin resistance causing increases in free fatty acids (FFA) [11].

Overall diet quality and nutritional composition have been shown to improve risk factors for CVD which can coincide with development and progression of prediabetes and T2D [15,16,17]. Therefore, understanding nutritional patterns, trends, and dietary factors within populations who have prediabetes and T2D, as well as their outcomes, is of importance. Protein is an essential macronutrient necessary for optimal growth, maintenance, and overall health of humans in all life stages [18]. The recommended dietary allowance (RDA) for protein for almost all healthy adults, and older individuals, in the USA, is 0.8 g (g)/kilogram (kg) body weight (bw) of protein, with no RDA established for individuals with prediabetes and T2D [19]. In addition to their biological roles and anabolic properties, dietary proteins can increase satiety, which can contribute to a reduced total energy intake, as well as increased thermogenesis causing an increase in energy expenditure [20]. Data from the National Health and Nutrition Examination Survey indicate fewer overweight and obese individuals meet the 0.8 g/kg bw protein dietary allowance daily, which is indicative of adverse health outcomes [21]. Protein intake above the RDA has received considerable interest with regards to favorable health outcomes related to reductions in body weight and fat mass (FM) when in the absence of increased total energy expenditure [21,22,23,24]. In addition to the favorable outcomes associated with body composition, protein consumption above the RDA has been shown to have beneficial effects on risk factors associated with CVD including blood pressure, cholesterol/lipid panel, and glycemic control in overweight or obese individuals [25,26,27]. A few studies have examined the beneficial effects of high protein diets acutely on glycemic control in individuals with T2D [22,28,29,30], however, there is inconclusive and insufficient evidence with regards to the association of the amount and type of protein with beneficial or adverse outcomes related to CVD risk in individuals who are diabetic or prediabetic [22]. There is a sparsity of studies with respect to long-term effects of protein intake on glycemic control [31,32]. Hence, to examine the associations between protein consumption and the risk factors associated with prediabetes and T2D is important. Therefore, the purpose of this cross-sectional study was to examine the relationships among protein consumption, glycemic control and indices of cardiovascular health in individuals with prediabetes and T2D. We hypothesized that consumption of greater than or equal to 1.0 g/kg bw dietary protein would be associated with the following: (a) improved indices of glucose control, for example, insulin resistance and insulin secretion; (b) lower fat mass (FM), body mass index (BMI), and waist-to-hip (WC/HC) ratio; and (c) higher lean mass (LM) in overweight and obese adults with prediabetes and T2D. Additionally, we hypothesized that the ratio of animal-to-plant protein would have a positive association with insulin sensitivity and lean mass in all sites assessed; furthermore, there would be an inverse association between the ratio of animal-to-plant protein and fasting blood glucose, insulin resistance, BMI, WC/HC, and fat mass in all sites assessed.

## 2. Materials and Methods

### 2.1. Participants

The data analyzed for this cross-sectional study were collected from screening and baseline study visits of two clinical trials conducted at Florida State University (Tallahassee, FL, USA) that had similar protocols and procedures. All experimental procedures conducted in these studies were approved by the Florida State University’s Institutional Review Board (HSC no. 2015.15638; 2017.20453) These trials are registered under clinicaltrials.gov (NCT02224365; NCT03272074). Men and women, between the ages of 45 and 75 years who were overweight or obese, with prediabetes or T2D were recruited from Tallahassee, FL and surrounding areas through campus and community advertisements via flyers, newspaper articles, and public events. Participants were eligible if their glycated hemoglobin (HbA1c) was ≥5.7% and their BMI was between 25.0 and 40.0 kg/m^2^ at the screening visit. Participants taking part in a weight loss program within the past 6 months; or who were heavy smokers (>20 cigarettes per day); heavy drinkers (>12 alcoholic drinks per week); or had a BMI <25.0 or >40.0 kg/m^2^; diagnosed CVD; uncontrolled hypertension (≥160/100 mmHg); and other active chronic diseases such as cancer, glaucoma, thyroid, kidney, liver or pancreatic disease were excluded from the study. Participants who met the inclusion criteria were considered for the study regardless of ethnicity and race. After initial pre-screening over the telephone, eligible participants were asked to come to the Human Performance Laboratory in the Department of Nutrition, Food and Exercise Sciences at Florida State University for an onsite screening and to provide written informed consent.

### 2.2. Study Overview

For this cross-sectional study, men and women between the ages of 45 to 75 years who were overweight or obese and who reported to having been diagnosed by a physician with prediabetes or T2D, were included and grouped based on their protein intake obtained from a three-day dietary log. Protein intake of less than 0.8 g/kg bw, ≥0.8 but <1.0 g/kg bw, and ≥1.0 g/kg bw were considered within this study to be below, meeting, and above the recommended levels of protein intake, respectively. After an initial screening via telephone, participants were asked to come to the clinical research facility for their screening visit. On the screening visit, after the participant gave their informed consent, measurements of their WC, HC, body weight, resting brachial blood pressure, and HbA1c using A1cNow^®^ were completed to confirm prediabetes or T2D. Questions on medical history and medication use were asked during this visit to confirm eligibility. If the participant was eligible, each participant met with the research personnel to learn how to accurately complete a three-day food record during the two weeks prior to the next study visit. Participants were given the three-day food record to take home and bring back to the clinical research facility on their next visit. Participants reported back to the clinical research facility for the visit where the research personnel reviewed the three-day food record for their three representative days (2 weekdays and 1 weekend day). During the study visit, between the hours of 7:00 and 10:00 a.m., after an overnight fast and 12 h after the abstinence of caffeine, each participant’s blood was drawn; anthropometrics including height, weight, WC, and HC were assessed; resting brachial blood pressure was taken; and dual-energy X-ray absorptiometry (DXA, GE Healthcare Lunar, Madison, WI, USA) scans were completed to assess body composition including lean mass (LM) and FM. Based on their typical intake from the three-day food record, participants were categorized into the three groups based on their quantity of protein intake.

### 2.3. Dietary Assessment and Determination of Animal-to-Plant Ratio

Three-day food records were used to determine the protein content of each participant’s diet and typical eating pattern. Participants were given instructions on how to fill out the three-day food records at the screening visit and were asked to choose three typical days to complete the three-day food records, two weekdays and one weekend day. The Food Processor software (Food Processor version 7.50, ESHA Research, Salem, OR, USA) was used to analyze each food item on the three-day food records. The averages of total energy, dietary fiber, total sugar, carbohydrate, fat, cholesterol, saturated, trans, monounsaturated, polyunsaturated fats, and protein consumption were determined from the three-day food records and calculated in gram/day (g/d) or milligram/day (mg/d) to determine the average intake for the three days.

The type of protein, animal versus plant, was also determined to assess the ratio of animal-to-plant protein, as well as total grams of animal versus plant protein. Animal protein sources included the following: meats (red meats, processed meats, poultry, and fish); eggs; and animal by-products (milk, cheese, and yogurt). Plant protein sources included the following: legumes, seeds, grains, nuts, nutritional yeast, soy products, and seitan. The Food Processor software cannot distinguish between animal or plant protein as separate protein sources. Thus, daily intakes were carefully and individually examined to determine if the protein source was from animal or plant sources to calculate the animal-to-plant protein ratio from the total gram intake.

### 2.4. Assessment of Anthropometrics and Blood Pressure

Height, weight, WC, and HC were measured at screening and study visits. The height of the participants (cm), after removal of their shoes, was measured using a wall-mounted stadiometer and their weight was measured using a digital scale (Seca Corporation, Chino, CA, USA). The BMI was calculated as weight (kg) divided by height (m^2^). The WC and HC of participants were measured using a Gulick fiberglass measuring tape with a tension handle (Creative Health Products, Inc., Ann Arbor, MI, USA) according to the World Health Organization protocol [33]. Briefly, the WC was measured in the horizontal plane midway between the lowest rib and the superior boarder of the right iliac crest. The HC was measured around the widest portion of the buttocks with the measuring tape parallel to the ground. For both of the WC and HC, the participants stood with their feet together and arms by their sides while evenly distributing body weight. Measurements were repeated twice and if the measurements were within 1 cm of each other, the average of the two measurements was calculated. Study personnel ensured that the tape fit comfortably but did not compress the skin. The WC/HC was calculated as WC divided by HC. Brachial blood pressure was measured using an automated blood pressure machine (Omron HEM-907XL Pro Healthcare, Vernon Hills, IL, USA) to determine each participant’s eligibility at the screening visit and measured at the study visit. Participants were asked to sit quietly for at least 10 min prior to taking blood pressure measurements with their feet uncrossed, while seated upright.

### 2.5. Statistical Analysis

Statistical analyses were performed with the IBM (International Business Machines) SPSS (Statistical Package for the Social Sciences) computer program (Version 25, Chicago, IL, USA) and all figures were created using Graphpad Prism (Version 7, San Diego, CA, USA). Descriptive statistics were calculated for all variables and included their corresponding mean ± standard error of the mean, and *p*-values ≤0.05 were considered statistically significant. Statistical differences for blood biomarkers and anthropometric measurements among below, meeting, and above the recommended levels of protein intake, were assessed using one-way analysis of variance (ANOVA). Additionally, total energy intake, as well as nutrients including fat, saturated fat, monounsaturated fat, polyunsaturated fat, trans fat, cholesterol, carbohydrate, fiber and total sugar were assessed using one-way ANOVA among individuals consuming below, meeting, and above the recommended protein levels. If a significant F-statistic was obtained, Fisher’s least significant difference post hoc test was used for pairwise comparisons. After determination of the protein source, the animal-to-plant protein ratio, total average grams of animal protein, and total average grams of plant protein were calculated and assessed for statistical differences in blood biomarkers and anthropometric measurements among below, meeting, and above the recommended levels of protein intake using one-way ANOVA. Similarly, if a significant F-statistic was obtained, Fisher’s least significant difference post hoc test was used for pairwise comparisons. The relationship between the mean intake of animal and plant protein and animal-to-plant protein ratio, within all groups and the outcome variables (insulin; glucose; homeostatic model assessment for insulin resistance (HOMA-IR); homeostatic model assessment for beta-cell function (HOMA-β); total-cholesterol; high-density lipoprotein (HDL)-cholesterol; triglycerides (TG); low-density lipoprotein (LDL)-cholesterol; oxidized-LDL (Ox-LDL); total, arm, leg, androidal, and gynoidal LM and FM; FM/LM ratio, body weight; systolic blood pressure; diastolic blood pressure; BMI; height; WC; HC; and WC/HC ratio) were evaluated by Pearson correlation coefficient (r). Magnitude of correlation coefficients was considered as very small to none (r < 0.1), small (0.1 ≤ r < 0.3), moderate (0.3 ≤ r < 0.5), large (0.5 ≤ r < 0.7), very large (0.7 ≤ r < 0.9), nearly perfect (0.9 ≤ r < 1.0), and perfect (r = 1.0) [34]. The sample size calculation was done using G* Power Software. Using a one-way ANOVA with fixed effects, based on a desired power of 0.80, a significance level set at 0.05, a large effect size (f = 0.42), and three total groups, a total sample size of 60 participants was needed.

## 3. Results

**Figure 1:**
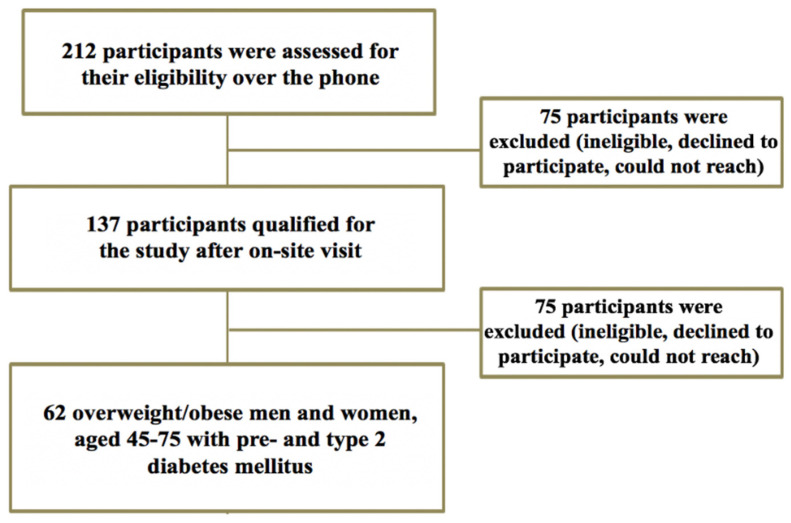
Study flowchart of recruitment and enrolment flowchart. Participants were excluded if they did not meet the inclusion criteria from questions over the phone or from on-site physical assessments and medical history questionnaires, declining participation, or not responding when contacted.

From the analysis of the two studies, a total of 212 participants were screened over the telephone and 137 of those participants were screened onsite for this cross-sectional study. Seventy-five participants who qualified were dropped from the study due to the inability to obtain three-day food records (*n* = 20), failure to contact (*n* = 38), and personal reasons (*n* = 17). Sixty-two participants (*n* = 40 females and *n* = 22 males) who met the inclusion criteria were included in the study. Clinical, laboratory, and statistical analyses were performed for the 62 participants who completed the screening and study visits. A flowchart of the study enrolment is presented in Figure 1.

Table 1 presents medication classification for each group, below, meeting, and above the recommended level of protein intake, respectively. There were no significant differences among groups for medication use, other than the participants in the recommended level of protein intake having significantly greater (*p* ≤ 0.05) usage of non-steroidal anti-inflammatory drugs (NSAID) as compared with the group who consumed below the recommended protein level.

### 3.1. Anthropometrics

**Figure 2:**
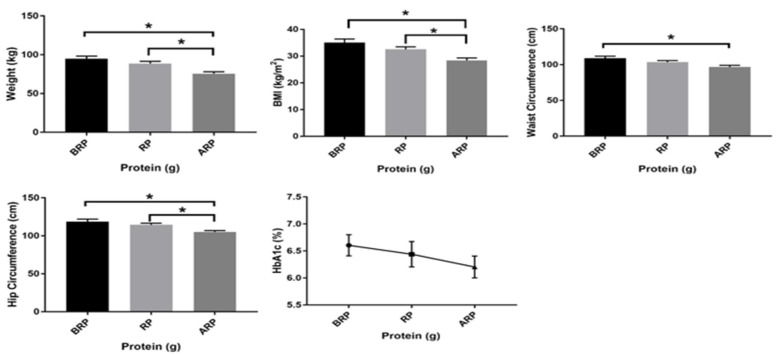
Among group differences in weight, body mass index (BMI), waist circumference, hip circumference, and hemoglobin A1c (HbA1c) of study participants below (*n* = 17), meeting (*n* = 22), and above (*n* = 23) the recommended-protein groups (below the recommended-protein group (BRP), the recommended-protein group (RP), and the above recommended-protein group (ARP), respectively). Values are reported as mean ± standard error of the mean (SEM). * Denotes significant differences among groups (*p* ≤ 0.05).

All anthropometric values were reported as mean ± standard error of the mean (SEM) (Table 2). Weight was significantly higher in participants who consumed below (95 ± 3.3 kg vs. 76 ± 2.4 kg, *p* ≤ 0.01) and the recommended (89 ± 2.9 kg vs. 76 ± 2.4 kg, *p* = 0.01) levels of protein as compared with above the recommended level of protein intake. BMI was significantly higher in participants who consumed below (35 ± 1.3 kg/m^2^ vs. 28 ± 0.94 kg/m^2^, *p* ≤ 0.01) and meeting the recommended (32 ± 0.92 kg/m^2^ vs. 28 ± 0.94 kg/m^2^, *p* = 0.04) level of protein intake as compared with above the recommended level of protein intake. HC was significantly greater in participants who consumed below and the recommended protein levels (119 ± 3.2 cm vs. 105 ± 1.8 cm, *p* ≤ 0.01; 115 ± 2.1 cm vs. 105 ± 1.8 cm, *p* ≤ 0.01) as compared with above the recommended protein level, respectively; WC was significantly greater in participants who consumed below the recommended protein levels (109 ± 2.7 cm vs. 97 ± 2.5 cm, *p* = 0.046) and the recommended protein levels (104 ± 2.3 cm vs. 97 ± 2.5 cm, *p* ≤ 0.01) as compared with above the recommended protein level. There were no significant differences in age, height, WC/HC ratio, HbA1c, SBP, and DBP. Although there were no statistically significant differences with HbA1c between groups, there were numerical and perhaps clinically relevant reductions in HbA1c levels amongst groups, as protein levels increased from below to above the recommended levels. Figure 2 shows the significant differences between weight, BMI, WC, HC, as well as HbA1c values amongst groups.

### 3.2. Differences in Nutrient Intake and Body Composition

Results from the analysis of the three-day food record showed that varying levels of protein contributed to significant differences in total energy, monounsaturated fat, polyunsaturated fat, cholesterol, carbohydrate, and fiber intake, as well as animal and plant protein consumption among groups shown in Table 3 (values were reported as mean ± SEM). Total energy intake was significantly lower in participants who consumed below the recommended protein level as compared with participants who were meeting (1627 ± 69 kcal vs. 2024 ± 1045 kcal, *p* = 0.02) and above the recommended protein levels (1627 ± 69 kcal vs. 2163 ± 125 kcal, *p* ≤ 0.01). Total fat consumption tended to be lower (68 ± 3.6 g vs. 86 ± 7.6 g, *p* = 0.052) in participants who consumed below the recommended protein level as compared with individuals who consumed above the recommended protein level. Mono- and polyunsaturated fat consumption were significantly lower in participants who consumed below the recommended protein level as compared with individuals who consumed above the recommended protein level, respectively (7.7 ± 1.3 g vs. 13 ± 1.7 g, *p* = 0.04; 4.6 ± 0.86 g vs. 8.1 ± 0.95 g, *p* ≤ 0.01). Polyunsaturated fat was also significantly lower for participants who consumed the recommended amount of protein as compared with individuals who consumed above the recommended protein level (4.2 ± 0.75 g vs. 8.1 ± 0.95 g, *p* ≤ 0.01). Cholesterol consumption was significantly lower with participants who consumed below the recommended amount of protein as compared with those meeting (243 ± 22 mg vs. 367 ± 38 mg, *p* = 0.049) and above the recommended levels (243 ± 22 mg vs. 411 ± 51 mg, *p* ≤ 0.01) of protein intake.

**Figure 3:**
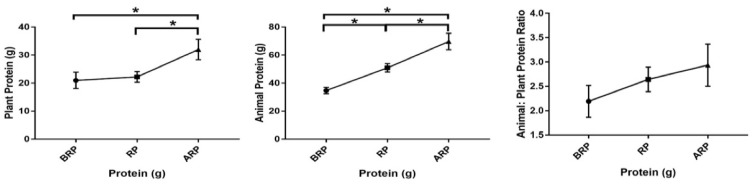
Among group differences in plant protein, animal protein, and animal-to-plant protein ratio intake from the three-day food record averages of study participants below (*n* = 17), meeting (*n* = 22), and above (*n* = 23) the recommended-protein groups (below recommended-protein group (BRP), recommended-protein group (RP), and above recommended-protein group (ARP), respectively). Values are reported as mean ± standard error of the mean (SEM). * Denotes significant differences among groups (*p* ≤ 0.05).

**Figure 4:**
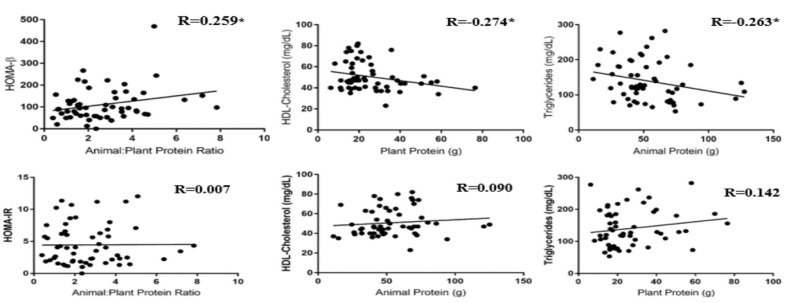
Correlations between homeostatic model assessment for insulin resistance (HOMA-IR) and homeostatic model assessment for beta-cell function (HOMA-β) with animal-to-plant protein ratio; HDL (high-density lipoprotein) cholesterol with animal and plant protein; triglycerides with animal and plant protein (*n* = 62). Values are reported as Pearson correlation coefficient (r). * Denotes significant differences among groups (*p* ≤ 0.05).

Carbohydrate intake was significantly lower in participants consuming below the recommended protein level as compared with individuals who consumed above the recommended protein level (190 ± 15 g vs. 253 ± 19 g, *p* = 0.02). Dietary fiber intake was significantly lower in the individuals who consumed below (16 ± 1.5 g vs. 23 ± 2.2 g, *p* = 0.012) and recommended (16 ± 1.5 g vs. 23 ± 2.2 g, *p* = 0.011) protein levels as compared with individuals who consumed above the recommended protein level. Intake of animal protein, similar to total protein intake, was significantly different among all groups (35 ± 2.3 vs. 51 ± 3.0 g vs. 70 ± 5.9 g, *p* ≤ 0.01). Plant protein intake was significantly lower in the groups that consumed below (21 ± 2.9 g vs. 32 ± 3.6 g, *p* ≤ 0.01) or recommended (22 ± 1.9 g vs. 32 ± 3.6 g, *p* = 0.02) protein levels as compared with individuals who consumed above the recommended protein level (Figure 3). There was a significant positive correlation (*p* = 0.03) between the total energy intake for individuals who met the recommended protein level and BMI. There was also a significant positive correlation (*p* = 0.02) between total energy intake and protein intake for individuals who met the recommended protein level (Table 4). There were no significant differences among groups in terms of saturated fat, trans fat, total sugar, or animal-to-plant protein ratio. Results from Pearson correlation analysis examining the relationship between outcome variables with animal-to-plant protein ratio, average animal protein intake, and average plant protein intake showed a significant positive correlation (*p* = 0.05) between HOMA-β and animal-to-plant protein ratio. Additionally, there were significant negative correlations between the average plant protein intake and HDL cholesterol levels (*p* = 0.041), as well as between the average animal protein intake and TG levels (*p* = 0.048) of participants (Figure 4).

**Figure 5:**
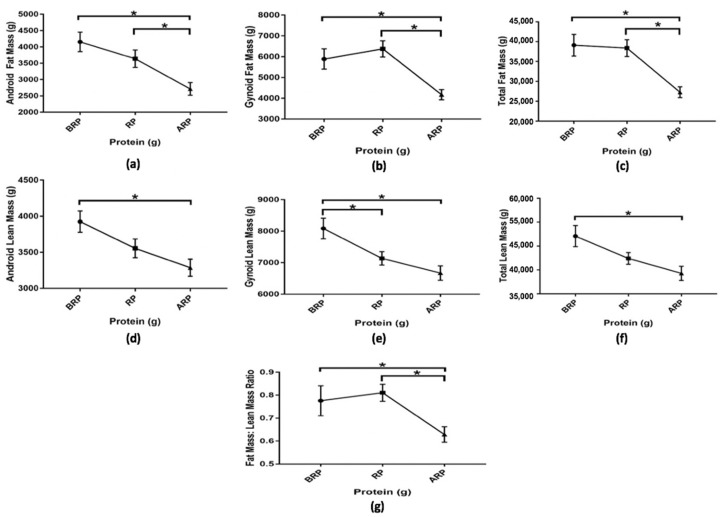
Among group differences in android fat mass (**a**), gynoid fat mass (**b**), total fat mass (**c**), android lean mass (**d**), gynoid lean mass (**e**), total lean mass (**f**), and fat mass: lean mass ratio (**g**) of study participants below (*n* = 17), meeting (*n* = 22), and above (*n* = 23) the recommended-protein groups (below recommended-protein group (BRP), recommended-protein group (RP), and above recommended-protein group (ARP), respectively). Values are reported as mean ± SEM. * Denotes significant differences among groups (*p ≤* 0.05).

Results from body composition analysis using DXA showed that the three categories of protein intake had significant differences for all parameters assessed. There was a similar pattern among groups for all parameters that assessed fat distribution (participant’s arms, legs, androidal region, gynoidal region, and total FM), whereas participants in the above recommended-protein group had significantly lower (*p* ≤ 0.01) FM in these areas as compared with participants in the below or the meeting recommended-protein groups. Furthermore, participants who consumed below the recommended protein level showed significantly higher (*p* < 0.05) LM in all sites assessed. The FM/LM ratio was significantly lower in participants who consumed above the recommended protein level as compared with participants who consumed below (0.78 ± 0.07 g vs. 0.63 ± 0.03 g, *p* = 0.03) and the recommended (0.81 ± 0.04 g vs. 0.63 ± 0.03 g, *p* ≤ 0.01) protein levels (Figure 5).

### 3.3. Differences in Lipid Profile, Glycemic Control, and Oxidized LDL

**Figure 6:**
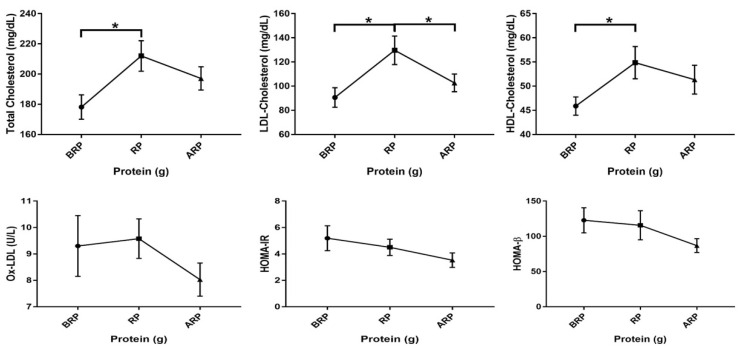
Among group differences in total cholesterol, low-density lipoprotein (LDL) cholesterol, high-density lipoprotein (HDL) cholesterol, oxidized LDL (Ox-LDL), homeostatic model assessment for insulin resistance (HOMA-IR), and homeostatic model assessment for beta-cell function (HOMA-β) of study participants below (*n* = 17), meeting (*n* = 22), and above (*n* = 23) the recommended-protein groups (BRP, RP, and ARP, respectively). Values are reported as mean ± standard error of the mean (SEM). * Denotes significant differences among groups (*p* < 0.05).

Values for lipid profiles, glycemic control, and Ox-LDL were stratified based on protein intake and are shown in Table 5 (values were reported as mean ± SEM). The mean total cholesterol concentration was significantly higher in participants in the recommended-protein group (212 ± 10 mg/dL vs. 178 ± 8.1 mg/dL, *p* = 0.01) than those in the below the recommended-protein group. Similarly, the mean LDL and HDL cholesterol concentrations were significantly higher in participants in the recommended-protein group (178 ± 8.1 mg/dL vs. 91 ± 8.1 mg/dL, *p* ≤ 0.01; 55 ± 3.3 mg/dL vs. 46 ± 1.9 mg/dL, *p* = 0.04, respectively) as compared with those in the below recommended-protein group. The LDL cholesterol was, similarly, significantly greater in participants who met the recommended protein intake as compared with individuals who consumed above the recommended protein level (130 ± 12 mg/dL vs. 103 ± 7.3 mg/dL, *p* = 0.04). The mean HOMA-IR value, tended to be significantly lower (3.5 ± 0.55 vs. 5.2 ± 0.94, *p* = 0.09) in those who consumed above the recommended protein level as compared with those who consumed below the recommended protein level. Figure 6 represents the mean values for HOMA-β, HOMA-IR, Ox-LD, as well as total LDL and HDL cholesterol.

## 4. Discussion

The findings of this cross-sectional study demonstrate that protein intake above the recommended levels in overweight and obese individuals with prediabetes and T2D is associated with favorable outcomes such as anthropometrics and body composition. We also observed that insulin resistance (determined from the HOMA-IR equation) tended to be lower in these individuals who had protein intake above the recommended level. Our findings indicate that individuals consuming the lowest amount of protein had the highest BMI, WC, and HC. In this cross-sectional study, individuals who consumed protein above the recommended level had lower total body weight, BMI, WC, and HC as compared with individuals who consumed below the recommended protein level or the recommended protein level; with no significant differences in age, height, WC/HC, HbA1c, and blood pressure. In a study by Park et al. [35], Korean adults 60 years and older who consumed the two highest quartiles of protein (both a median intake of greater than 1 g/kg/d of total protein) from both plant and animal sources had significantly lower total body weight, BMI, and WC.

The observations related to anthropometric measurements, in the present study, are consistent with other studies that have found that individuals with and without metabolic disease who consumed above the RDA have lower BMI values than individuals consuming below the RDA level [24,25,26,27,29,36,37,38,39,40]. However, this has not been established in individuals with prediabetes or T2D, comparing individuals below, meeting, or above the RDA, or examining the relationship between outcome variables with animal versus plant protein sources. In the present study, the participants with higher BMI, HC, and WC were also the participants who reported the lowest total caloric intake. However, the group with protein consumption of 0.8 to less than 1.0 g/kg bw protein was the only group in which there was a positive correlation between total energy intake and BMI, as well as between total energy intake and protein intake. Therefore, this implies that this was the only group of individuals who were perhaps not underreporting their caloric intake. In a pooled analysis of three prospective studies [31], the association between protein consumption and the incidence of T2D from men and women was examined. Unlike our findings, the total energy intake of men and women in the lower tertile of protein intake consumed higher total energy as compared with men and women in the highest tertile of protein intake. A possible reason for the differences in our results could be due to our stratification of groups based on protein consumption by g/kg bw as opposed to protein consumption based on the percent of an individual’s total energy intake. From this study, when groups are stratified by percentage of total energy intake their protein intake becomes 14.5, 15.4, and 17.9% for below, meeting and above the recommended-protein groups, respectively. Nonetheless, similar to our study, in a population-based cross-sectional study by Li et al. [41], the association between dietary protein intake and the prevalence and risk factors related to T2D, specifically dietary fat, was assessed in men and women from Northeast China. In that study, individuals were stratified into tertiles based on protein intake (averaged g/d) from food frequency questionnaires and similar upward trends for monounsaturated fat, polyunsaturated fat, and cholesterol consumption were observed in individuals who consumed the highest amount of protein as compared with those who were in the lowest tertile [42,43]. With regards to the trend of individuals decreasing fat and carbohydrate consumption with increases in protein intake, the results from our study conflict with the results in the literature where underreporting and stratification of protein consumption based on g/kg/bw as opposed to a percentage of total energy from protein could have contributed to this discrepancy observed.

Interestingly, in our cross-sectional study, the participants who consumed the highest amount of protein also consumed nearly twice as much animal protein as compared with plant protein. This is anticipated because most individuals consume dietary protein as either meat, fish, or poultry, or as dietary supplements, in the USA [19]. Plant protein was inversely associated with serum HDL cholesterol concentrations in our study participants, suggesting that animal protein could be beneficial for improving HDL cholesterol. This is in agreement with other studies [44,45] that have reported that animal protein was associated with increases in HDL cholesterol more than plant protein. In our study, the increase in animal protein consumption was also shown to be associated with lower TG levels. The findings of a prior cross-over design study, in part, agree with our findings. In that study, healthy normolipidemic men were given animal or plant protein for 21 days and assessed for lipid values [44]. They found that HDL cholesterol was increased only from the animal protein consumption with no changes in LDL cholesterol or TG levels between groups. However, our findings were from a small cross-sectional study and the magnitudes of the correlations that were significant were small. Therefore, future studies are needed to evaluate animal versus plant protein intake with lipid biomarkers in a much larger study with a similar population. A possible reason why total LDL and HDL cholesterol concentrations were higher in the recommended-protein group could be due to factors, aside from total protein consumption, such as saturated or trans fats intake. Participants who consumed above the recommended protein level did not have values that were significantly different for total and HDL cholesterol as compared with the participants who consumed below or recommended protein levels, which perhaps indicates that the protein intake in the recommended group was not the reason for the higher cholesterol levels observed. There are limited studies [26,36,38,39] that have assessed the long-term effects of protein consumption on lipid biomarkers; additionally, there have not been consistent results, especially in populations with metabolic diseases [46,47]. In a longitudinal study of men and women by Bahadoran et al. [46], dietary intake, anthropometrics, and lipid panels were assessed at baseline and after three years. Individuals in the highest quartile of protein consumption had the lowest total cholesterol levels, regardless of the amount of carbohydrates in the diet. Further research is needed on protein source effects on lipid profile.

Although our results did not indicate significant between group differences in HbA1c, this observation is in accordance with a meta-analysis conducted by Dong et al. [22]. In that meta-analysis, the effects of high protein diets (>20% of total energy) were examined with outcome measures including glycemic control and blood pressure in randomized controlled trials, at least four weeks in duration, in overweight or obese individuals with T2D. Results from the pooled analysis indicated that, in addition to significant weight loss (from 4.2 to 4.7 kg), the high protein diets as compared with the low protein diets (15–20% of total energy) were associated with significant reductions in HbA1c, by 0.52%. Although not statistically significant, individuals who consumed below the recommended protein level had 0.50% (7.8% difference) greater HbA1c, and those who met the recommended protein level had 0.30% (3.2% difference) greater HbA1c, as compared with those who were in the above recommended-protein group. A cross-sectional study of men and women by Green et al. [48] also reported similar findings. Results from our study showed that insulin and glucose levels, as well as HOMA-IR and -β, were not different among protein intake groups, indicating that insulin resistance was similar among groups. Further studies are needed to examine glycemic indices and various levels of protein intake within this population.

In the present study, individuals in the below recommended-protein group had higher LM, as well as FM in all sites as compared with individuals who consumed above the recommended protein level. This tendency was also seen in certain sites for individuals who consumed the recommended protein level. Individuals who consumed above the recommended protein level had the lowest FM/LM ratio, indicating a higher percentage of lean mass as compared with individuals who consumed below the recommended protein level. This indicates that individuals who were in the below the recommended-protein group were more overweight and obese as compared with the other groups with a greater protein intake. Results for the group that consumed the highest amount of protein had the lowest body fat in all sites assessed, which is in agreement with a cross-sectional study by Green et al. [48] with similar results. Increasing dietary protein above the recommended protein intake can be especially beneficial in older adults with metabolic diseases, such as T2D, due to increased protein intake potentially limiting the loss of muscle that is commonly seen within this population [49,50,51]. In a cross-sectional study by Ezeh et al. [52], the authors indicated that a higher FM/LM ratio was independently associated with fasting insulin levels, HOMA-IR, and HOMA-β. Our study did show that individuals who consumed above the recommended protein level had numerically the lowest HbA1c percentages, HOMA-IR, blood glucose, and insulin levels. In this cross-sectional study, there was a tendency for HOMA-IR to be lower in those who consumed the greatest amount of protein. In support of our observations, the findings of a nine-month randomized clinical study by de Luis et al. [53], indicted that obese individuals who consumed a high protein/hypocaloric diet had more pronounced reductions in anthropometrics, insulin levels, and HOMA-IR as compared with individuals who consumed a standard hypocaloric diet. In a six-week randomized controlled feeding trial, anthropometrics, body composition, and indices of glycemic control were assessed in adults with elevated TG/HDL ratios who received either a calorie-restricted high protein diet or control diet [54]. In addition to increases in LM, participants who consumed the hypocaloric high protein diet had a 26% improvement in HOMA-IR. Longer-term studies are needed to further examine the role of increased protein intake in reducing insulin resistance.

Some limitations of this study include the design of the study which was a cross-sectional, non-interventional study, where causality could not be directly determined. Interventional and long-term clinical trials, with a larger sample size, are needed within this population to examine the effects of increased protein consumption, as well as animal and plant sources of protein on indices of glycemic control and cardiovascular health. Another common limitation is that of inaccuracies with questionnaires that involve self-reporting dietary intake; in addition, longer time periods (more than three days) of recording dietary intake should be done for future studies. Research personnel involved with this study reviewed the three-day food records during the participants’ visits to ensure the information was completed correctly. DXA scans were used in this study for assessment of body composition, which have been shown to overestimate LM in obese individuals with the DXA scanner model used in this study [55]. Additionally, medication use was not controlled for (other than no insulin usage) in the stratified groups for blood pressure, hyperglycemic, lipid lowering, NSAID, and thyroid medications. Additionally, future studies should also examine amino acid components of diets to further examine amino acid composition on outcomes measured from this study.

## 5. Conclusions

Lifestyle modifications, such as dietary changes, can decrease the risk of development of chronic diseases including T2D. While there are recommendations for the general population, there are no established recommendations for protein intake for individuals with prediabetes and T2D. Findings from this study suggest that protein intake above the recommended daily intake (greater than 1.0 g/kg bw), in overweight and obese individuals with prediabetes and T2D is associated with lower insulin resistance, in addition to lower BMI, WC, FM, and FM/LM ratio as compared with individuals consuming less than 1.0 g/kg bw. This study could not confirm relationships between protein intake and total, LDL, and HDL cholesterol concentrations. This is consistent with the general understanding that dietary cholesterol, commonly present in higher protein diets, does not significantly alter circulating levels of cholesterol in the fasted state. The overall findings of this cross-sectional study indicate that higher protein intake, especially from animal sources, was associated with better β-cell function and lower TG concentrations. Furthermore, large-scale interventional studies are needed to examine the dose-dependent effects of protein, as well as plant versus animal protein sources and their effects on factors associated with glycemic control and cardiovascular health within populations with prediabetes and T2D.

## Figures and Tables

**Table 1:** List of medication categories and usage.

Variable: Medications	Below Recommended Protein (*n* = 17)	Meeting Recommended Protein (*n* = 22)	Above Recommended Protein (*n* = 23)	Total (*n* = 62)
Antihypertensive: *n* (%)	11 (65)	9 (41)	10 (43)	30 (48)
Hyperglycemic: *n* (%)	9 (53)	8 (36)	6 (26)	23 (37)
Lipid Lowering: *n* (%)	6 (35)	10 (45)	7 (30)	23 (37)
Hypothyroid: *n* (%)	3 (18)	2 (9)	2 (9)	7 (11)
NSAID: *n* (%)	0 (0) ^b^	7 (32)	3 (13)	10 (16)

Values are expressed as *n* (%) for each group. Abbreviation: NSAID, non-steroidal anti-inflammatory drug. ^b^ Denotes significance differences (*p* ≤ 0.05) from meeting the recommended level of protein intake.

**Table 2:** Demographic information and anthropometrics.

Variable:	Below Recommended Protein (*n* = 17)	Meeting Recommended Protein (*n* = 22)	Above Recommended Protein (*n* = 23)	Total (*n* = 62)
Male/Female (*n*/*n*)	7/10	7/15	8/15	22/40
Pre-DM/ T2D (*n*/*n*)	8/9	16/6	19/4	43/19
Age (years)	60 ± 1.7	63 ± 1.4	62 ± 1.4	62 ± 0.8
Height (cm)	164 ± 1.7	165 ± 1.4	164 ± 1.5	165 ± 0.9
Weight (kg)	95 ± 3.3 ^c^	89 ± 2.9 ^c^	76 ± 2.4	86 ± 1.9
BMI (kg/m^2^)	35 ± 1.3 ^c^	33 ± 0.9 ^c^	28 ± 0.9	32 ± 0.7
Waist Circumference (cm)	109 ± 2.7 ^c^	104 ± 2.3 ^c^	97 ± 2.5	103 ± 1.5
Hip Circumference (cm)	119 ± 3.3 ^c^	115 ± 2.1 ^c^	106 ± 1.8	112 ± 1.5
Waist-to-Hip Ratio (cm)	0.92 ± 0.01	0.91 ± 0.02	0.92 ± 0.02	0.92 ± 0.01
HbA1c (%)	6.7 ± 0.20	6.4 ± 0.23	6.2 ± 0.20	6.4 ± 0.12
SBP (mmHg)	132 ± 4.3	133 ± 4.0	130 ± 2.9	131 ± 2.1
DBP (mmHg)	80 ± 3.0	79 ± 1.8	77 ± 1.8	78 ± 1.2

Values reported as mean ± standard error of the mean (SEM). ^c^ Denotes significant differences from the above recommended level of protein intake. Abbreviations: BMI, body mass index; HbA1c, hemoglobin A1c; SBP, systolic blood pressure; DBP, diastolic blood pressure; Pre-DM, prediabetic; T2D, type 2 diabetes. ^c^ Denotes significant differences (*p* ≤ 0.05) from the above recommended level of protein intake.

**Table 3:** Nutrient intake.

Variable:	Below Recommended Protein (*n* = 17)	Meeting Recommended Protein (*n* = 22)	Above Recommended Protein (*n* = 23)	Total (*n* = 62)
Energy Intake (Kcal)	1627 ± 69	2024 ± 105 ^a^	2163 ± 125 ^a^	1967 ± 67
Fat (g)	68 ± 3.6 ^d^	83 ± 5.6	86 ± 7.6	80 ± 3.6
Saturated Fat (g)	23 ± 1.9	25 ± 2.4	27 ± 2.9	25 ± 1.5
Monounsaturated Fat (g)	7.7 ± 1.3 ^c^	9.2 ± 1.7	12.7 ± 1.7	10.1 ± 1.0
Polyunsaturated Fat (g)	4.6 ± 0.86 ^c^	4.2 ± 0.75^c^	8.1 ± 0.95	5.8 ± 0.55
*Trans* Fat (g)	1.1 ± 0.27	1.1 ± 0.27	1.3 ± 0.34	1.2 ± 0.17
Cholesterol (mg)	243 ± 22	367 ± 38 ^a^	411 ± 51 ^a^	349 ± 25
Carbohydrates (g)	190 ± 15 ^c^	234 ± 19	253 ± 19	228 ± 11
Dietary Fiber (g)	16 ± 1.5 ^c^	16 ± 1.5^c^	23 ± 2.2	19 ± 1.1
Total Sugar (g)	84 ± 11	91 ± 14	91 ± 8	89 ± 6
Protein (g)	59 ± 3.5 ^b, c^	78 ± 2.2 ^a, c^	97 ± 4.1 ^a, b^	80 ± 2.7
Animal Protein (g)	35 ± 2.3 ^b, c^	51 ± 3.0 ^a, c^	68 ± 5.9 ^a, b^	53 ± 3.0
Plant Protein (g)	21 ± 2.9 ^c^	22 ± 1.9 ^c^	32.4	26 ± 1.8
Animal/Plant Protein Ratio	2.2 ± 0.33	2.6 ± 0.25	2.9 ± 0.43	2.6 ± 0.21

Values reported as mean ± standard error of the mean (SEM). ^a^ Denotes significant differences from below the recommended protein level, ^b^ denotes significance differences from meeting the recommended protein level, and ^c^ denotes significant differences from above the recommended protein level. ^d^ Tends to be significantly (*p* ≤ 0.1) different as compared with above the recommended protein level. Significance was set at *p* ≤ 0.05.

**Table 4:** Correlations between body mass index, total body fat percentage, and total protein intake with total energy intake.

Variables:	Total Energy Intake for BRP (kcals)	Total Energy Intake for MRP (kcals)	Total Energy Intake for ARP (kcals)
BMI (kg/m^2^)	0.236	0.460 *	0.190
Total Body Fat (g)	0.302	0.416	0.161
Protein Intake (g)	0.372	0.505 *	0.433

Values reported as Pearson correlation coefficient (r). Abbreviations: BMI, body mass index. * Denotes significant (*p* < 0.05) correlations between variables. Correlations were done from the 3-day average of total protein and energy intake of study participants of study participants below (*n* = 17), meeting (*n* = 22), and above (*n* = 23) the recommended protein groups (below recommended-protein group (BRP), recommended-protein group (RP), and above recommended-protein group (ARP), respectively).

**Table 5:** Fasting lipid profile and glycemic control.

Variable:	Below Recommended Protein (*n* = 17)	Meeting Recommended Protein (*n* = 22)	Above Recommended Protein (*n* = 23)	Total (*n* = 62)
Blood Glucose (mg/dL)	119 ± 5.7	121 ± 6.9	116 ± 5.5	119 ± 3.5
Total Cholesterol (mg/dL)	178 ± 8.1	212 ± 10 ^a^	197 ± 7.7	197 ± 5.3
LDL-Cholesterol (mg/dL)	91 ± 8.1	130 ± 12 ^a, c^	103 ± 7.4	109 ± 5.8
HDL-Cholesterol (mg/dL)	46 ± 1.9	55 ± 3.3 ^a^	51 ± 3.0	51 ± 1.7
Triglycerides (mg/dL)	142 ± 13	147 ± 12	128 ± 12	138 ± 7.1
Insulin (uIU/dL)	17 ± 2.7	15 ± 1.8	13 ± 1.5	15 ± 1.1
Oxidized-LDL (U/L)	9.3 ± 1.2	9.6 ± 0.75	8.0 ± 0.63	8.9 ± 0.47
HOMA-IR	5.2 ± 0.94	4.5 ± 0.62	3.5 ± 0.55 ^d^	4.3 ± 0.40
HOMA-β	123 ± 18	116 ± 20	87 ± 9.8	106 ± 9.4

Values reported as mean ± SEM. Abbreviations: LDL, low-density lipoprotein; HDL, high-density lipoprotein; HOMA-IR, homeostatic model assessment for insulin resistance; HOMA-β, homeostatic model assessment for beta-cell function. ^a^ Denotes significant differences from below the recommended level of protein intake and ^c^ denotes significant differences from above the recommended level of protein intake. ^d^ Tends to be significantly (*p* ≤ 0.1) different as compared with below the recommended level of protein intake.
